# Comparing the efficacy of fluconazole and cryotherapy Versus cryotherapy alone on treating cutaneous leishmaniasis: a triple-blind randomized clinical trial

**DOI:** 10.1186/s12879-024-09211-5

**Published:** 2024-03-20

**Authors:** Ahmad Reza Parhizkar, Mehdi Sharafi, Susan Mansuri, Maryam Hadibarhaghtalab, Sima Afrashteh, Hossein Fatemian, Mahsa Rostami Chijan

**Affiliations:** 1 Noncommunicable Diseases Research Center, Fasa University of Medical Sciences, Fasa, Iran; 2 Infectious and Tropical Diseases Research Center,Hormozgan Health Institute, Hormozgan University of Medical Sciences, Bandar Abbas, Iran; 3 Molecular Dermatology Research Center, Shiraz University of Medical Sciences, Shiraz, Iran; 4 Department of Biostatistics and Epidemiology, Faculty of Health and Nutrition, Bushehr University of Medical Sciences, Bushehr, Iran; 5 Student Research Committee, Shiraz University of Medical Sciences, Shiraz, Iran; 6 Department of Persian Medicine, Fasa University of Medical Sciences, Fasa, Iran

**Keywords:** Fluconazole, Cryotherapy, Placebo, Leishmania, Leishmaniasis

## Abstract

**Objective:**

Cutaneous Leishmaniasis (CL) is one of the highly prevalent endemic diseases in the Middle East. The disease is a complex skin infection imposing a heavy burden on many developing countries. This study aimed to evaluate the impact of adding oral fluconazole to topical cryotherapy on the treatment efficacy and time to achieve complete recovery of CL lesions.

**Method:**

This triple-blind randomized clinical trial included 52 participants with CL. Participants were allocated to receive either weekly cryotherapy with liquid nitrogen and oral fluconazole at a dose of 6 mg/kg daily at a maximum of 400 mg for 6 weeks as the interventional arm or weekly cryotherapy with liquid nitrogen plus the placebo for the same period of 6 weeks as the control arm.

**Results:**

Fifty-two eligible participants enrolled the study, with a CL lesion count of 1 to 8 (mean 1.96), and served as the interventional (*n* = 28) and control (*n* = 24) arms. The trend of the mean surface area of the lesions was significantly decreasing in both arms (*P* < 0.001), with no statistically significant difference between arms (*P* = 0.133) or all assessed time point pairwise comparisons (*P* > 0.05). There was no significant difference between the treatment arms in terms of the end-point recovery status (*P* = 0.491) or the frequency of post-treatment secretion (*P* = 0.437). No adverse effect was observed.

**Conclusion:**

Despite a slightly higher reduction in the lesion surface in the cryotherapy and fluconazole treatment arm, the addition of fluconazole did not provide statistically significant therapeutic value to cryotherapy in the treatment of CL. However, with adjustment for the initial lesion size, the efficacy of the regimen in the interventional arm was more pronounced, though it was still insignificant.

## Introduction

Leishmaniasis is a disease caused by a group of intracellular protozoans of the genus *Leishmania* that can be transmitted to mammals, especially humans [1]. The disease is transmitted by the bite of sandflies, especially *Phlebotomus* species, in the Old World [2]. According to the recent World Health Organization (WHO) report, about 700,000 to 1 million new cases are added annually [1]. Among the types of leishmaniasis, cutaneous leishmaniasis (CL) is the most prevalent disease [3], with a concerning reporting rate of 20–33% annual new cases reported to WHO [1]. Since 2010, when the WHO established the Leishmaniasis Special Committee, there has been increased attention to epidemiological and interventional studies on leishmaniasis [4].

The management of CL differs from region to region and is primarily based on local experience-based evidence. Several treatment modalities have been used for CL, including topical treatments, systemic therapy, cryotherapy, photodynamic therapy, etc. [5]. Topical and local treatments are the preferred modality in treating most CL patients, comprising the clinically simple Old World Cutaneous Leishmaniasis (OWCL) lesions and the localized New World Cutaneous Leishmaniasis (NWCL) lesions caused by the *Leishmania* species that are not associated with mucosal involvement [6]. Systemic therapy is required for some *Leishmania* species that can cause mucocutaneous involvement. The gold standard for systemic therapy remains systemic antimonial [7]. Cryotherapy, which involves the use of extreme cold to destroy abnormal tissue, is effective in the treatment of OWCL when combined with topical Juniperus excelsa M. Bieb cream [8].

Among different treatments for CL, the WHO has suggested the use of pentavalent antimonial drugs as the gold standard therapy [9]. However, pentavalent antimonial drugs may cause various side effects and toxicities, including musculoskeletal pain, headache, nausea, asthenia, cardiotoxicity, hepatotoxicity, nephrotoxicity, and pancreatitis [10]. In addition, these drugs are contraindicated among patients with pneumonia, myocarditis, hepatitis, nephritis, pregnant or breast-feeding mothers, etc.; hence, as these agents cannot be used in all patients, many studies have been conducted to investigate the efficacy and safety of various topical and systemic therapies. However, reported treatments do not usually provide consistent results and many treatment modalities may fail in a presenting patient. For example, systemic meglumine antimoniate with a standard dose of 20 mg per Kg per day given intramuscularly over 2 weeks shows about an 81% cure rate for *L. major* and 48% for *L. tropica*. Noticeably, given the lower efficacy obtained for the *L. tropica* CL lesions, several studies have implemented the combination therapies, which have been proved to increase the treatment efficacy [11].

One of the most useful combination regimens to treat CL is weekly intralesional Glucantime® plus cryotherapy. However, since CL is endemic in many regions, exerts a substantial financial burden on the healthcare system for prevention and treatment, and there still exists a large knowledge gap about treatment modalities with high cure rates, there is a demand for identifying more efficacious treatment strategies. As both fluconazole, an oral medication, and liquid nitrogen, a topical therapy, are shown to be effective for CL [12, 13], we designed and conducted a placebo-controlled randomized clinical trial (RCT) to evaluate the efficacy and safety of adding oral fluconazole to cryotherapy among CL patients.

## Methods and materials

### Study design and participants

**Fig. 1:**
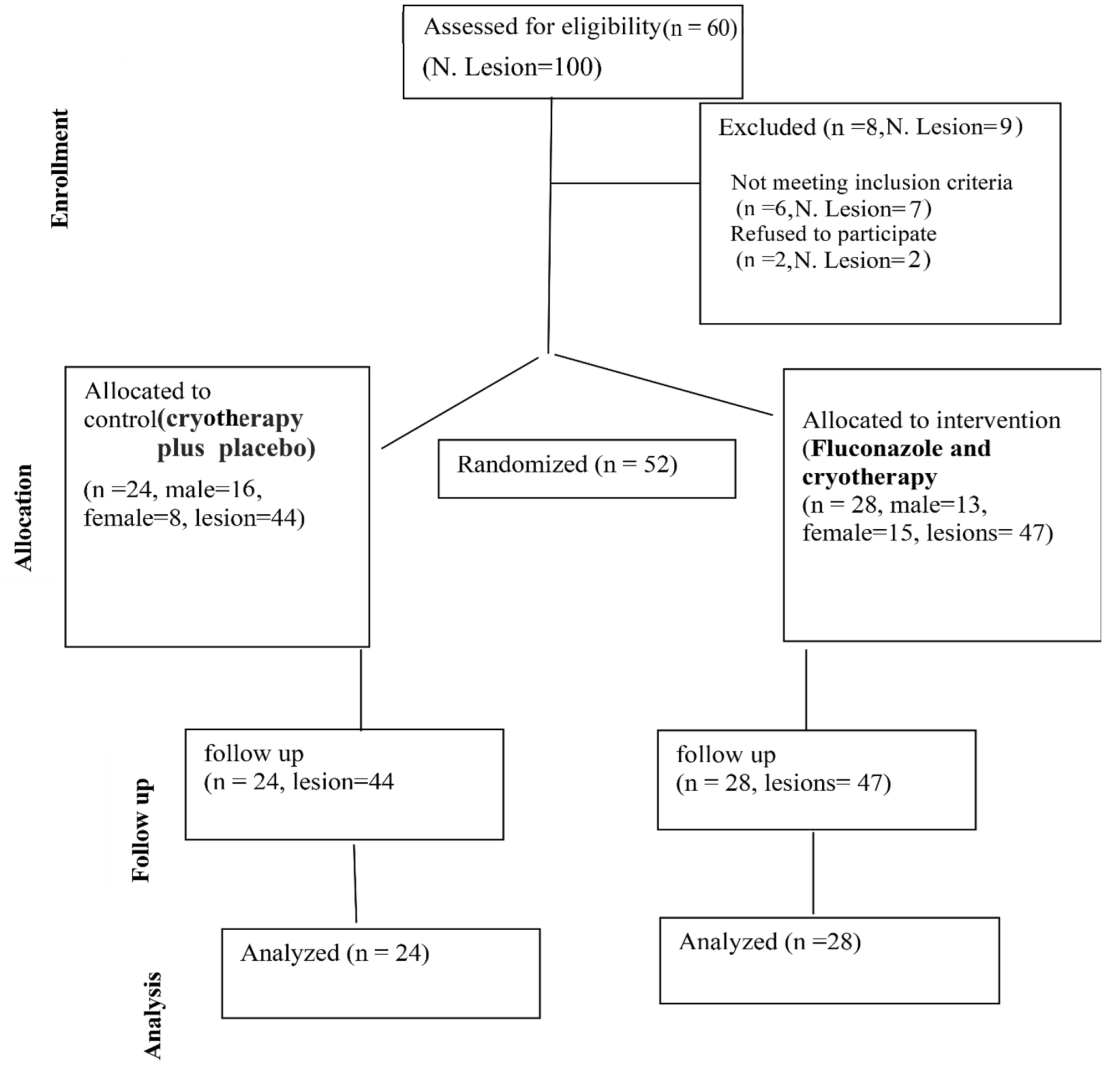
CONSORT diagram for the flow of participants through each stage of the trial

This triple-blinded RCT was conducted among subjects with CL who attended the Fatemieh referral OPD clinic, affiliated with Fasa University of Medical Sciences in southern Iran, during 2015–2016. The study intended to include two 30-patient arms. The CONSORT diagram of each stage is shown in Fig. 1.

#### Inclusion and exclusion criteria

Criteria for entering the study were: weight above 10 kg, disease initiation-to-referral duration of less than 1 month, age over 5 years, and a positive smear. Exclusion criteria included pregnancy and breastfeeding, being a traveller, the presence of lesions on the ear and face, lesion count of more than 10, history or presence of kidney or heart disease, lupoid and sporotrichoid cases, participants with immunodeficiency, HIV, and diabetes.

#### Randomization and blinding

Participants who fulfilled the inclusion criteria, entered the study after signing the written informed consent form, and their demographic characteristics were recorded, including name, gender, age, weight, place of residence, occupation, underlying diseases, and CL characteristics. Participants were randomized into either the interventional arm (cryotherapy plus fluconazole) or the control arm (cryotherapy plus the placebo), using the random block design of AB and BA. Fluconazole and placebo were placed in two identical packages (label A or B), that were available to the program administrators. Only, the pharmacist was aware of the packages’ content, while the investigators, the participants, and the data analyst were blinded. To blind the intervention, both fluconazole and the placebo capsules were packaged by a pharmacist in the form of 50 mg capsules of similar appearance. Each capsule might contain 50 mg of fluconazole or 50 mg of the placebo (Roasted corn flour).

The executor physician allocated the intervention A or B according to the random sequence list, generated by the statistician. The principal investigator was a dermatologist who recorded and documented each session’s information for the participants. All measurement sessions were carried out using the same measurement devices at a fix location.

At baseline, the recorded CL characteristics included as following: [1] lesion shape: If the lesion was localized, its type was determined. In addition, if the lesion exhibited central ulceration and oozing and was secreting discharge, it was defined as a wet type, and it was considered dry vice versa; [2] history of infection and treatment: The new case was a case that presented for the first time and had never been treated; [3] Diameters of lesion and ulcer (mm); [4] Number of lesions and their location. The present study did not use polymerase chain reaction (PCR) to identify the *Leishmania* species, and the wet and dry type are used only as descriptive measure. In addition to the limited recourses, our previous publication using PCR evaluation in the region where the participants were recruited, showed that almost all of the CL cases causative specie (137 out of 138) in the Fasa district are *L. major* and *L. tropica* is seen only in travellers from other areas [14], which is one of the exclusion criteria in the present study; therefore, the possibility of mixed infections of *L. major* and *L. tropica*– the varying intrinsic therapeutic responses– would be negligible.

### Interventions

Participants in the interventional arm received weekly cryotherapy with liquid nitrogen and oral fluconazole at a dose of 6 mg/kg daily, allowing a maximum of 400 mg, for 6 weeks. The control therapy consisted of weekly cryotherapy with liquid nitrogen plus placebo, for 6 weeks. Each capsule was sufficient for 8 kg body weight in both arms; therefore, the number of capsules consumed varied based on the participant’s weight. However, for participants heavier than 64 kg, no more than 8 capsules were given. Cryotherapy involved applying liquid nitrogen via a cotton swab for 10–25 s until the lesion and its’ adjacent 1–2 mm normal tissue appeared frozen, through two thaw and freeze cycles, in both arms. Moreover, participants were recommended not to dress the lesions and to keep them dry, as well as to use mupirocin ointment to prevent bacterial infection.

### Assessments

#### Efficacy assessments

To obtain and compare the treatments’ efficacy, participants’ lesion characteristics, comprising diameters of the lesion in mm, lesion type, and the extent of lesion induration, were recorded in both arms at the baseline and six weekly reassessments, as well as a post-treatment follow-up reassessment 4 weeks after cessation of therapy. To measure the diameter of the lesions two vertical and horizontal vectors were measured at the largest part, using a flexible ruler from the beginning of redness on one side to the end of redness on the other side. The lesion’s surface area was measured indirectly by a mathematical formula (*S = π.(vd).(hd)/4)*. The similar principle was used to measure the lesion’s induration. That is, the beginning of the thickening and hardening of the tissue is considered the starting point for measurement. It should be noticed that since induration is the most important factor indicating complete recovery, which is the last component being improved, and measuring surface area incorporates both the vertical and horizontal diameter of the lesion, we decided to use the mathematically calculated lesion’s induration surface area. In addition, this method takes advantage of more summarized and less complicated data presentation.

In addition, at the follow-up reassessment, the lesion recovery status was evaluated and obtained as [1] complete recovery (i.e., clearance of induration and complete re-epithelialization of the lesions) [2], relative recovery (i.e., decreased induration surface by more than 50% and having a negative smear), and [3] no improvement (decreased induration surface less than 50% or having a positive smear).

#### Safety assessments

Due to the possibility of renal and hepatic toxicity related to fluconazole, liver and kidney function tests were performed at the beginning of the study before starting treatment and 3 weeks after the start of the treatment. If complications were observed, the allocated intervention was stopped, the participants were dropped out from the study, and rescue therapy was initiated. Additionally, any adverse event that could be attributed to the interventions was recorded.

#### Rescue therapy

In case of any safety concern during the trial, the participant was excluded from the trail and was allocated rescue therapy. In addition, 4 weeks after treatment cessation, participants were re-evaluated for the stability of the results observed at the end of the study or the changes towards improvement or deterioration. This is because of the participants’ rights to be treated completely not for the purpose of this study protocol. If a participant had not reached a complete recovery at the 4th post-trial week, standard treatment protocol of the National Program of Leishmaniasis Elimination of Iran would have been applied, i.e., weekly intralesional injection of Glucantime® plus cryotherapy.

### Statistical analysis

At the end of the study, collected data were sent for statistical analysis using Statistical Package for the Social Sciences (SPSS; version 22.0; IBM Corp., Armonk, NY, USA). After analysing the data and at the time of interpretation, the regimen used in each arm was revealed to the research team. Descriptive statistical methods, including frequency (%) and mean (standard deviation), were employed to report the results. The independent sample T-test and the Pearson’s Chi-square test were utilized for pairwise comparison at each time point. In addition, the general linear model (GLM) was used to analyse the trends of outcome’s repeated measurements. In this model, firstly, the Mauchly’s test of sphericity was checked, in which if the sphericity assumption was not fulfilled (*P* < 0.001), the Greenhouse-Geisser correction for within-subject effects was reported for each arm. Furthermore, the Pillai’s trace was utilized to compare the arms for their trends of outcome’s repeated measurements.

#### Sample size

The comparison proportion for the independent two-group formula was used to calculate the sample size: n = (Z_α_/2 + Z_β_) ² * (p1(1-p1)/n1 + p2(1-p2)/n2) / (p1-p2) ². According to the study of Alrajhi et al. [15], considering the type one error (α = 0.05) and power (1-β = 0.80), and the probability of success groups (p1 = 0.34, p2 = 0.79), and the two-tailed test, a minimum sample size of 44 participants was calculated, which then increased to 52 to improve the power of the study.

### Ethical considerations

The study was approved by the ethics committee of Fasa University of Medical Sciences (ID: IR.FUMS.REC.1394.2) and was registered in the Iranian Registry of Clinical Trials (IRCT) with registration number IRCT2014112320052N1. Participants were enrolled in the study after giving their written informed consent. Consent was taken from the legal guardian (Father mostly) of the children under the age of 18 years.

## Results

### Baseline data

Fifty-two participants aged between 5 and 59 years (mean age of 29.81 ± 15.48 years) with a mean CL lesion count of 1.96 ± 1.4, ranged 1–8, allocated to receive either fluconazole plus cryotherapy (*n* = 28) or placebo plus cryotherapy (*n* = 24). Additionally, the interventional and control arms consisted of 49 and 51 lesions, respectively. Most of the participants (78.84%) had one or two lesions, and almost all of the lesions were wet lesions (91 lesions) except one participant in the control arm who had two dry lesions. Twenty-nine (55.8%) participants were from the city and 23 (44.2%) were from the village, with no significant correlation with lesion count (*P* = 0.8). No significant difference was noted between the two arms in terms of age, sex, weight, lesion count, and living place (*P* > 0.05) (Table 1).

**Table 1:** Baseline characteristics of the two arms

Variable	Total number of participants (*n* = 52)	Cryotherapy and fluconazole (*n* = 28)	Cryotherapy and placebo (*n* = 24)
Sex			
Male Female	29 (55.7%) 23 (44.2%)	13 (46.4%) 15 (53.6%)	16 (66.7%) 8 (33.3%)
Residence			
City Village	29 (55.7%) 23 (44.3%)	19 (67.85%) 9 (32.15%)	10 (41.6%) 14 (58.4%)
Age, year) mean ± SD)	29.81 ± 15.48	30.21 ± 17.07	29.33 ± 13.73
Weight, kg (mean ± SD)	64.02 ± 21.97	60.22 ± 21.79	68.47 ± 21.80
Type of lesion			
Wet Dry Missed	49 (94.2%) 1 (1.96%) 2 (3.84%)	27 (96.5%) 0 (0%) 1 (3.5%)	22 (91.6%) 1 (4.2%) 1 (4.2%)
Number of lesions			
1 2 3 > 3 Mean ± SD Total	25 (48.07%) 16 (30.76%) 6 (11.53%) 5 (9.61%) 1.96 ± 1.4 100	17 (60.71%) 7 (25%) 2 (7.14%) 2 (7.14%) 1.75 ± 1.45 49	8 (33.33%) 9 (37.5%) 4 (16.66%) 3 (12.5%) 2.20 ± 1.31 51
Horizontal diameter of the ulcer, cm			
Range Mean ± SD	0.00–3.00 0.86 ± 0.78	0.00–3.00 0.81 ± 0.78	0.00–3.00 0.91 ± 0.78
Vertical diameter of the ulcer, cm			
Range Mean ± SD	0.00–3.00 0.68 ± 0.64	0.00–3.00 0.67 ± 0.67	0.00–2.00 0.68 ± 0.62
The surface of the ulcer, cm^2^			
Range Mean ± SD	0.00–23.55 3.19 ± 5.17	0.00–23.55 3.16 ± 5.51	0.00–17.90 3.22 ± 4.88
Horizontal diameter of the lesion, cm			
Range Mean ± SD	0.60–4.50 2.15 ± 0.97	0.60–4.50 2.34 ± 1.05	0.60–4.30 1.96 ± 0.85
Vertical diameter of the lesion, cm			
Range Mean ± SD	0.50–3.30 1.78 ± 0.68	0.50–3.30 1.87 ± 0.73	0.60–3.30 1.69 ± 0.63
The surface of the lesion, cm			
Range Mean ± SD	0.94–44.56 13.78 ± 10.46	0.94–42.39 15.81 ± 11.45	1.32–44.56 11.83 ± 9.11

The mean horizontal and vertical diameters of the ulcers were 0.81 ± 0.78 and 0.67 ± 0.67 cm in the interventional arm and 0.91 ± 0.78 and 0.68 ± 0.62 cm in the control arm, which yielded the calculated mean surface area of 3.16 ± 5.51 cm^2^ and 3.22 ± 4.88 cm^2^, respectively. There were no significant differences between the two arms in terms of these measurements (*P* > 0.05). Moreover, the mean horizontal and vertical diameters of the lesion (i.e., induration) were 2.34 ± 1.05 cm and 1.87 ± 0.73 cm in the interventional arm and 1.96 ± 0.85 cm and 1.69 ± 0.63 cm in the control arm, which yielded the calculated mean surface area of 15.81 ± 11.45 and 11.83 ± 9.11 cm^2^, respectively. It is notable that a nearly significant larger horizontal diameter of the induration in the interventional arm resulted in a larger surface area of the lesion; that is, lesions in the intervention arm were larger compared to the control arm, although it was not statistically significantly (Table 1).

### Efficacy

The results of repeated measures within each arm indicated a significant decreasing trend in the mean surface area of the lesions in both arms (*P* < 0.001). When comparing arms for mean surface area of the lesion at each assessment time point, there is no significant difference for all pairwise comparisons (*P* > 0.05). Moreover, the trends of decrease in the mean surface area of the lesions were not significantly different between the interventional and control arms (*P* = 0.133) (Table 2). Apparently, the statistically significant decrease in lesions’ size was firstly appeared in week 6 when the last episode of treatment was given. Therefore, it shows that designing this study based on the 6-week treatment protocol was acceptable. The calculated difference in lesion size for each week compared to the baseline size is presented in Table 2.

**Table 2:** Trend and comparison of the mean (± SD) surface area of the lesion in two arms during the course of treatment

Arms	Week							
	1	2	3	4	5	6	Post-treatment	P ^1^
Cryotherapy and fluconazole	14.83 ± 10.76	16.64 ± 10.86	15.51 ± 11.34	15.11 ± 11.80	11.68 ± 9.31	7.57 ± 7.66	4.27 ± 5.84	< 0.001
Cryotherapy and placebo	12.07 ± 8.92	14.84 ± 11.95	14.72 ± 10.56	14.73 ± 11.39	13.73 ± 13.15	9.35 ± 9.02	6.23 ± 8.62	< 0.001
P ^2^	0.164	0.433	0.721	0.870	0.371	0.291	0.188	0.133 ^3^
Mean difference with baseline, cm^2^ Value 95% confidence interval Standard error P ^4^	-0.076 -1.019, 0.867 0.475 0.874	2.263 0.631, 3.895 0.822 0.007	1.645 0.108, 3.183 0.775 0.036	1.454 -0.195, 3.104 0.831 0.083	-0.781 -3.028, 1.466 1.132 0.492	-5.066 -7.238, -2.894 1.094 < 0.001	-8.302 -10.852, -5.751 1.285 < 0.001	-

^1^ Repeated Measures Greenhouse-Geisser to assess the trend in the outcome for each arm

^2^ Independent sample T-test to compare two arms in each time point

^3^ GLM to compare the arms for the trend in the outcome

^4^ Independent sample T-test to compare with baseline

**Fig. 2:**
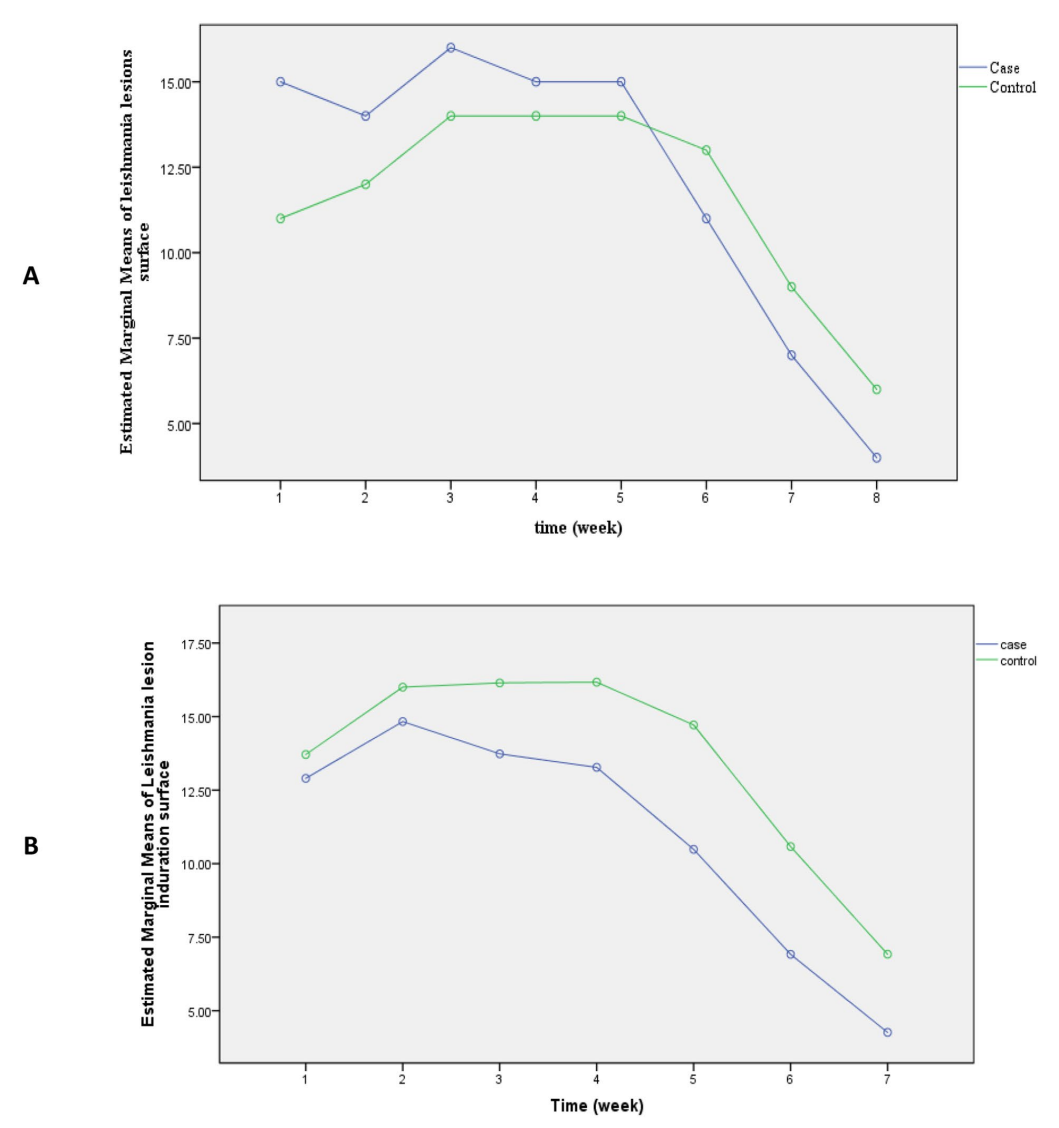
Estimated marginal means of CL lesions surface in two arms (**A**) without [time 1 represents the baseline measurement before starting the trial] and (**B**) with model corrected for the induration surface of lesions before starting the trial [time 1 represents the first measurement after starting the trial]

To compare the arms for trends of change in lesion size, we plotted the estimated marginal means of repeated measures (Fig. 2A). It shows no significant reduction during the first 4 weeks of treatment; then after, the curve slope down and it continues even after the last intervention episode, representing continuous healing despite discontinuation of treatment. It is noteworthy that the interventional arms’ curve crosses that of the control arm by the week 4 of treatment; that is, the reduction trend of the interventional arm suddenly falls below the control arm and it continues onward. Moreover, because the baseline lesion sizes varied, we corrected the model by considering this variable as a covariate (Fig. 2B). This model is also confirmed that the 4th episode of intervention was the game-changer in the healing trend of CL lesions. Additionally, the baseline size of lesions interferes with the reduction size of the induration. Despite the lack of statistically significant difference between the interventional and control arms, the interventional arm falls below the control arm at a relatively constant distance, probably indicating that the experimental intervention might be more effective for more advanced larger lesions than the control intervention.

The final assessment, conducted 4 weeks after the cessation of treatments, showed that 53.1% and 41.2% of lesions had completely recovered in the interventional and control arms, respectively. In addition, 22.4% and 27.5% of lesions were failed to heal in the interventional and control arms, respectively. Generally, there was no significant difference between the two arms in terms of the end-point recovery status (*P* = 0.491). Moreover, the frequency of post-treatment secretion was not statistically different between the interventional and control arms (10.2% versus 4.3%, *P* = 0.437) (Table 3).

**Table 3:** Comparison of the frequencies of lesions’ end-point recovery status and post-treatment secretion in two arms

Variable	Cryotherapy and fluconazole (*n* = 49)	Cryotherapy and placebo (*n* = 51)	Total (*n* = 100)	P ^2^
Recovery status Complete recovery Relative recovery No improvement	26 (53.1%) 12 (24.5%) 11 (22.4%)	21 (41.2%) 16 (31.3%) 14 (27.5%)	47 (47%) 28 (28%) 25 (25%)	0.491
Post-treatment secretion Positive Negative	5 (10.2%) 44 (89.8%)	2 (4.3%) ^1^ 44 (95.7%)	7 (7.4%) 88 (92.6%)	0.437

^1^ Five missed data.

^2^ Pearson’s Chi-square test

### Safety

All participants completed the treatment period, and no adverse effects attributed to the intervention were observed. Additionally, none of the participants exhibited any deteriorating changes in liver and kidney function tests or required rescue therapy.

## Discussion

Our study showed that treatment with oral fluconazole and cryotherapy, as well as cryotherapy plus placebo, resulted in a reduction in the surface area of lesions. Notably, the reduction observed in the oral fluconazole and cryotherapy arm was somewhat more pronounced. Also, the frequency of complete and relative recovery in the interventional arm was higher than in the control arm. However, no significant difference was observed between the two arms. Given the limited number of studies addressing the cure rate based on *Leishmania* species, additional evidence is essential to conclusively determine the efficacy of azoles against each Leishmania species.

Cryotherapy is a local therapeutic modality in the management of CL with variable reported efficacy [16]. It is accompanied by a painful sensation and has several mild to serious complications including susceptibility of the wounds to infections. At the same time, evidence exists that combination therapy can improve the treatment duration and outcome [17].

Interest in using azoles for leishmaniasis was revived after the report by Berman, demonstrating the activity of ketoconazole against *Leishmania* species in macrophage culture [18]. The results of the present study showed that no participants had to exit the study due to the adverse effects of fluconazole or cryotherapy. In line with this, Michelerio et al. reported no significant adverse effects in pediatric OWCL treated with oral fluconazole [19]. Alrajhi et al. evaluated the effect of fluconazole for the treatment of CL caused by *L. major* and showed that side effects were mild and similar in both arms (fluconazole arm and placebo arm) [15]. Prates et al. who investigated the efficacy of fluconazole in the treatment of CL due to *L. brasiliensis*, reported the side effects were similar in both arms (fluconazole arm and Glucantime® arm) [20]. Fluconazole has been successfully used in children with CL caused by *L. major* and *L. tropica.* Furthermore, no serious side effects were observed [19, 21]. Fluconazole has a longer half-life and greater concentrations in skin tissues than other azoles with lower toxicity. Hepatotoxicity is the most frequently reported side effect, and it usually manifests as conjugated hyperbilirubinemia or abnormal liver enzymes. Systemic fluconazole is highly tolerable in lactating and pregnant women [22]. Generally, no specific complications and side effects have been mentioned for the use of topical formulation of the drug, except for itchiness in several studies [23], although clinical studies do not appear to be enough to conclude. In the present study, no hepatotoxicity was observed in the treatment arm.

The results of repeated measures analysis within each arm indicated a significant decrease in the mean surface area of the lesion. Also, the lesion size (surface) significantly declined after introducing the intervention in both arms (time effect) but the difference was not significant when both arms were compared to each other. However, the reduction rate of the lesion surface area in patients treated with fluconazole was slightly greater than in patients treated with the placebo.

Consistent with the results of the present study, in the study of Parvizi et al., lesion count, duration of lesions, baseline vertical diameter size, baseline horizontal diameter size, and the baseline area of lesions showed no statistically significant differences between both arms [8]. In Saudi Arabia, Larbi et al. conducted a double-blind RCT comparing clotrimazole 1% and miconazole 2% topical creams for the treatment of CL lesions over 30 days [24]. The results showed that in the miconazole arm, no lesions had a complete recovery and only 30.5% of the lesions showed a decrease in size. On the other hand, in the clotrimazole arm, 15.7% of lesions were completely improved and 47.2% showed a size reduction, which confirmed that clotrimazole is significantly more effective than miconazole. Topical ketoconazole did not show a significant difference regarding effectiveness and improvement of lesions compared to placebo cream [23]. The cure rates were similar among the fluconazole, ketoconazole, and itraconazole arms [25]. Contrary to the results of our study, Mussi et al. compared the effect of topical fluconazole with topical paromomycin in BALB/c mice infected with *L. major*, showing a significantly higher effectiveness for the paromomycin arm [26]. The anti-leishmanial effects of azole antifungals are due to the inhibition of cytochrome P-450 mediated 14α-demethylation of lanosterol in fungi, which blocks ergosterol synthesis leading to the accumulation of 14α-methyl sterols. The inhibition of sterol biosynthesis causes leishmaniasis growth cessation [27]. In vitro studies regarding the effects of azoles on sterol biosynthesis have reported that, for most *Leishmania* species, itraconazole was slightly more inhibitory than ketoconazole, and fluconazole was much less inhibitory than the other azoles [13].

Based on the results of the present study, as indicated by the observed difference in lesion size, a statistically significant change first appeared in week 6, coinciding with the completion of the 6-week treatment protocol. This suggests that designing this study based on a 6-week treatment protocol was acceptable for conducting RCTs of this nature.

The duration of therapy is a crucial factor to consider. Some investigators have advocated the use of higher doses of fluconazole for a shorter period (6 weeks) [28, 29]. For instance, in a previously cited study, fluconazole was used for only 28 days [20]. The results of another study by Veraldi et al. showed that a longer duration of fluconazole treatment did not lead to significant side effects nor laboratory abnormalities. Previous data on the use of fluconazole for 6 weeks to treat OWCL caused by *L. major* have shown high cure rates with daily doses of 200 mg (79%) [15]. In line, a study by Emad et al. reported increased efficacy with a doubled dose of fluconazole (400 mg per day) [23]. Therefore, the dose of fluconazole chosen for the present study was based on this rationale.

In the study of Alrajhi et al., the median time to healing was 8.5 weeks for the fluconazole treatment arm, which was shorter than that of the control arm [15]. Also, none of the independent variables, including the size and number of lesions and their locations, had a significant effect on the healing process [15]. However, in our study, although the frequency of complete and relative recovery was higher in the interventional arm than in the control arm, no significant difference was observed. In addition, the type of treatment did not show any obvious effect on the amount of secretion.

Several studies have demonstrated the in vitro and in vivo efficacy of azole antifungals in the treatment of CL [30, 31]. The first clinical use of fluconazole in leishmaniasis was against kala-azar, with 0% definite cure rate [32]. Consistent with the results of our study, Sousa et al. reported a cure rate of 89% among 28 participants treated with fluconazole [33]. Similarly, Alrajhi et al. found that fluconazole resulted in complete healing for 79% of participants with CL caused by *L. major*, compared to 34% in the placebo arm [15]. In Brazil, a case series involving 28 participants with confirmed leishmaniasis caused by *L. braziliensis*, showed varying cure rates (75 to 100%) depending on the fluconazole dosage regimen: Eight participants received 5 mg/Kg/day with a cure rate of 75%, 14 participants received 6.5 mg/Kg/day with a cure rate of 92.8% and six participants received 8 mg/Kg/day with a cure rate of 100% [27]. However, in Prates et al. study, fluconazole administered orally at a dose of 6.5–8 mg/kg/d for 28 days was found to be ineffective in a high-transmission area for *L. braziliensis* [34].

Regarding imported leishmaniasis cases, oral fluconazole has shown varying and not always satisfying cure rate in RCTs involving *L. braziliensis*, which is responsible for mucocutaneous disease in the NWCL [20]. The cure rates for *L. major* infection, the cure rate varied between studies, ranging from 44.4% [25] to 79% [15]. The observed differences in cure rates among studies might be attributed to factors such as the dose regimen used, the Leishmania species involved, and variations in study methodology. Specifically, the final efficacy rate for *L. braziliensis* was 49%, based on an analysis involving only 138 participants. Based on only one or two studies with efficacy data for each of the other *Leishmania* species, the cure rate ranged from 15% for participants with *L. tropica* to 89% for *L. mexicana* [19]. These observations reinforce the need for further research to determine the actual efficacy of azole treatments and caution against relying solely on non-comparative and methodologically fragile studies to assess the usefulness of this class of drugs.

A study conducted by Frajzadeh et al. is interesting in this regard. They compared the combination of cryotherapy with oral terbinafine and cryotherapy with systemic meglumine antimoniate in the treatment of leishmaniasis. In their study, although the trend of healing was more slowly in the terbinafine arm, there was no significant difference between the two arms at the end. They used terbinafine at a double dose i.e., 125 mg/kg/d (for less than 20 kg body weight), 250 mg/kg/d (20–40 kg body weight), 500 mg/kg/d (for more than 40 kg body weight) [35]. Therefore, doubling the dose of antifungals may offer a clue to increase the efficacy of these regimens in treating leishmaniasis and could open new avenues for future research.

A study by Asilian et al. evaluated the effect of intralesional meglumine antimoniate and cryotherapy on leishmaniasis in three arms: intralesional meglumine plus cryotherapy, intralesional meglumine alone, and cryotherapy alone. The results showed that the combination arm achieved a 90.9% cure rate, while both monotherapy arms showed approximately a 55% cure rate. Thus, they stated the combination of Intralesional meglumine antimoniate plus cryotherapy is more effective than each of meglumine antimoniate or cryotherapy alone [36]. Another study by Noor et al. compared a combination of meglumine antimoniate plus cryotherapy to cryotherapy alone and showed similar better efficacy in the combination arm.

Jowkar et al. evaluated the added benefit of topical nitric oxide 3% to cryotherapy on CL and they reported no significant increase in the efficacy of treatment [37].

Fekri et al. conducted a study to compare the efficacy of co-administration of topical niosomal dapsone gel and intralesional injection of Glucantime® with cryotherapy plus intralesional injection of Glucantime® in CL. They had two arms of intralesional Glucantime® plus cryotherapy and intralesional Glucantime® plus niosomal dapson gel. They showed that there is no significant difference between the two arms. However, one cannot compare cryotherapy to dapson gel, because the effect of Glucantime® might be the dominant factor and compensate for the difference between cryotherapy and niosomal dapson gel [38].

This study had limitations. Firstly, lesion characteristics were not equally distributed in the interventional and control arms. Secondly, use of different doses of fluconazole was not evaluated, as varying doses of the medicine may yield different effects in the treatment of the disease. Future studies should consider evaluating the effects of different doses of fluconazole in treating the disease. It is also suggested to evaluate the effect of other azoles in the treatment of leishmaniasis. Thirdly, another aspect that should be highlighted is the effect of leishmaniasis ulcer sizes at baseline on the outcomes. In this study, we did not initially consider this point in study design; nonetheless, we compensated this drawback with an adjustment at the time of analysis, as it stood out as depicted in the figures.

Our study highlights the importance of encouraging research for new effective therapies with oral drugs for CL treatment, preferably in RCTs, stratified by geographic region of study and by *Leishmania* species. Also, it is prudent to think of multimodal or multi-drug therapy in the treatment of such disease in future research to develop more effective treatments with fewer side effects that could potentially replace antimoniate drugs.

## Conclusions

Despite a slightly higher reduction in lesion surface in the cryotherapy and fluconazole treatment arm, fluconazole added no statistically significant therapeutic value to cryotherapy in the treatment of CL. However, with adjustment for the initial lesion size, the efficacy of the regimen in the interventional arm was more pronounced, though it was still insignificant. Larger RCTs are warranted for the assessment and optimization of the presented treatment strategy in different regions and *Leishmania* species.

## Data Availability

The data that support the findings of this study are available from the corresponding author.
